# Supplementary material for: Optimization of nitrogen and carbon removal with simultaneous partial nitrification, anammox and denitrification in membrane bioreactor
Source: R Soc Open Sci. 2020 Sep 9;7(9):200584. doi: 10.1098/rsos.200584 (PMC7540762; doi:10.1098/rsos.200584)
Supplement: Supplementary figures and Tables [file rsos200584supp1.doc]

**Supplementary figures**

**Figure captions**

**Fig.1S.** Multiple-response optimization.

**Fig.2S.** The nitrogen and COD removal performance during stable operation of SNAD.

**Fig.3S.** The periodic change of DO concentration in different phases.

**Tables captions**

**Table 1S** Composition of synthetic wastewater in batch tests

**Table 2S** The design of RSM and obtained results

**
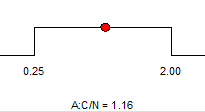

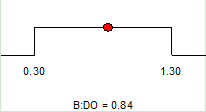

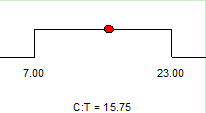

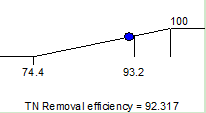

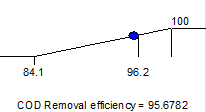
**

**Fig.1S.** Multiple-response optimization.

**Fig.2S.** The nitrogen and COD removal performance during stable

operation of SNAD.

**Fig.3S.** The periodic change of DO concentration in different phases.

**Table 1S** Composition of synthetic wastewater in batch tests

| constiuents | AerAOB | AnAOB | DNB_a_ | DNB_b_ |
| --- | --- | --- | --- | --- |
| NH_4_^+^-N | 200 | 100 | 0 | 0 |
| NO_2_^-^-N | 0 | 132 | 100 | 0 |
| NO_3_^-^-N | 0 | 0 | 0 | 100 |
| COD | 0 | 0 | 100 | 100 |

**Table 2S** The design of RSM and obtained results

| Run | X_1_  C/N | X_2_  DO | X_3_  T_ae_ | Response 1  TIN removal efficiency % | Response 2  COD removal efficiency % |
| --- | --- | --- | --- | --- | --- |
| 1 | 0.25 | 0.3 | 15 | 77.3 | 88.3 |
| 2 | 2 | 0.8 | 23 | 84.6 | 90.9 |
| 3 | 1.13 | 0.8 | 15 | 93.2 | 96.2 |
| 4 | 1.13 | 0.3 | 7 | 74.4 | 92.2 |
| 5 | 1.13 | 0.8 | 15 | 93.2 | 96.2 |
| 6 | 2 | 0.3 | 15 | 74.4 | 90.5 |
| 7 | 2 | 0.8 | 7 | 77.9 | 91.1 |
| 8 | 1.13 | 0.8 | 15 | 93.2 | 96.2 |
| 9 | 1.13 | 1.3 | 23 | 81.3 | 91.1 |
| 10 | 1.13 | 0.3 | 23 | 79.4 | 88.4 |
| 11 | 1.13 | 1.3 | 7 | 78.5 | 89.7 |
| 12 | 1.13 | 0.8 | 15 | 88.3 | 93.4 |
| 13 | 2 | 1.3 | 15 | 75.3 | 92.4 |
| 14 | 1.13 | 0.8 | 15 | 93.2 | 96.2 |
| 15 | 0.25 | 0.8 | 23 | 87.3 | 85.4 |
| 16 | 0.25 | 0.8 | 7 | 86.1 | 85.3 |
| 17 | 0.25 | 1.3 | 15 | 88.3 | 84.1 |
